# Gallbladder tuberculosis as an incidental calcified mass in an elderly male: a case report

**DOI:** 10.1093/jscr/rjag123

**Published:** 2026-03-07

**Authors:** Ho Tran, Kim H Nguyen, Thanh P C Nguyen, Phuoc T Bui

**Affiliations:** Department of General Surgery, Binh Dan Hospital, 371 Dien Bien Phu Street, Ban Co Ward, Ho Chi Minh City 700000, Vietnam; General Surgery Department, University of Medicine and Pharmacy at Ho Chi Minh City, 217 Hong Bang Street, Cho Lon Ward, Ho Chi Minh City 700000, Vietnam; Department of Liver Tumor, Binh Dan Hospital, 371 Dien Bien Phu Street, Ban Co Ward Ho Chi Minh City 700000, Vietnam; Department of Liver Tumor, Binh Dan Hospital, 371 Dien Bien Phu Street, Ban Co Ward Ho Chi Minh City 700000, Vietnam; Department of Liver Tumor, Binh Dan Hospital, 371 Dien Bien Phu Street, Ban Co Ward Ho Chi Minh City 700000, Vietnam; Department of General Surgery, Pham Ngoc Thach University of Medicine, 02 Duong Quang Trung Street, Hoa Hung Ward, Ho Chi Minh City 700000, Vietnam

**Keywords:** gallbladder tuberculosis, laparoscopic cholecystectomy, gallbladder calcification, tuberculous lesion, hepatobiliary tuberculosis

## Abstract

Gallbladder tuberculosis (GBT) is exceptionally rare and may closely mimic gallbladder carcinoma. We report a 71-year-old male with a history of successfully treated pulmonary tuberculosis who was asymptomatic and incidentally found on screening ultrasonography to have a calcified mass at the gallbladder fundus. Contrast-enhanced computed tomography (CT) demonstrated a 2 × 4 cm calcified fundal lesion with focal wall thickening, raising strong suspicion of malignancy. Laparoscopic cholecystectomy with intraoperative frozen section was performed. Despite severe inflammatory adhesions suggestive of invasive cancer, frozen section and final histopathology confirmed caseating granulomatous inflammation consistent with GBT, and surgery was limited to simple cholecystectomy. The postoperative course was uneventful, and the patient was referred for antituberculous therapy. GBT should be considered in the differential diagnosis of calcified gallbladder masses to avoid overtreatment.

## Introduction

Abdominal tuberculosis accounts for approximately 1%–3% of extrapulmonary tuberculosis cases and is considered uncommon. Hepatobiliary tuberculosis represents an even rarer manifestation, with an estimated prevalence of around 1% [1]. Among these, gallbladder tuberculosis (GBT) is an exceptionally rare clinical entity, with no clearly established incidence even in tuberculosis-endemic regions [2]. Its clinical presentation is nonspecific and often mimics gallbladder carcinoma [3], making preoperative diagnosis based solely on clinical and radiological findings particularly challenging [2]. This challenge becomes even more pronounced when the lesion presents as a calcified gallbladder mass, a radiological feature that itself raises strong suspicion for malignancy [4, 5]. We present a case of GBT presenting as an incidental calcified fundus mass initially suspected to be malignancy.

## Case report

A 71-year-old Asian male presented for routine health screening and was referred after ultrasonography revealed a calcified mass at the gallbladder fundus. He denied abdominal pain, fever, anorexia, weight loss, and jaundice and reported no systemic symptoms of active tuberculosis. His history included pulmonary tuberculosis diagnosed 6 years earlier, treated with a standard 6-month regimen, with confirmed cure. Physical examination was unremarkable.

**Figure 1:**
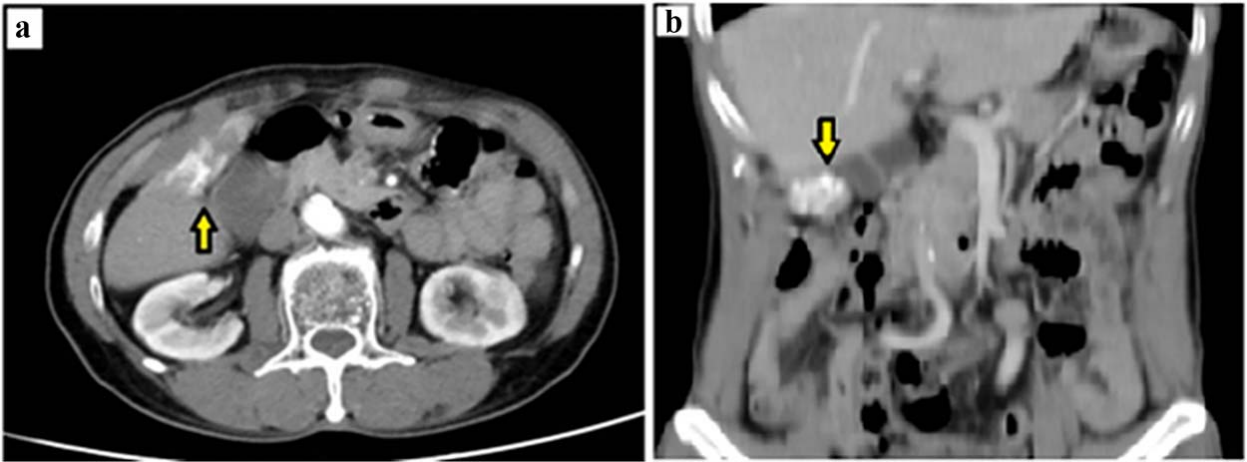
Preoperative contrast-enhanced CT demonstrating a calcified gallbladder fundus lesion suspicious for malignancy. (a) Axial CT shows a calcified mass at the gallbladder fundus with adjacent wall thickening (arrow). (b) Coronal CT reconstruction highlights the calcified lesion involving the gallbladder wall (arrow).

Laboratory tests showed normal liver function. White blood cell count was 8.61 k/μl (lymphocytes 22.1%). Tumor markers (CEA, CA 19-9, and AFP) were within normal limits. Chest radiography showed no active pulmonary lesions. Contrast-enhanced abdominal computed tomography (CT) demonstrated a bilobed gallbladder. In the distal (fundus) compartment, a 2 × 4 cm calcified mass was identified with adjacent wall thickening up to 4 mm (Fig. 1a and b). Multiple gallstones were present, with the largest measuring 11 mm. A gallbladder tumor with concomitant cholelithiasis was suspected. Because malignancy could not be excluded, laparoscopic cholecystectomy with intraoperative frozen section was planned, with preparedness to proceed to open radical cholecystectomy with hepatic wedge resection and hepatoduodenal lymphadenectomy if carcinoma was confirmed.

**Figure 2:**
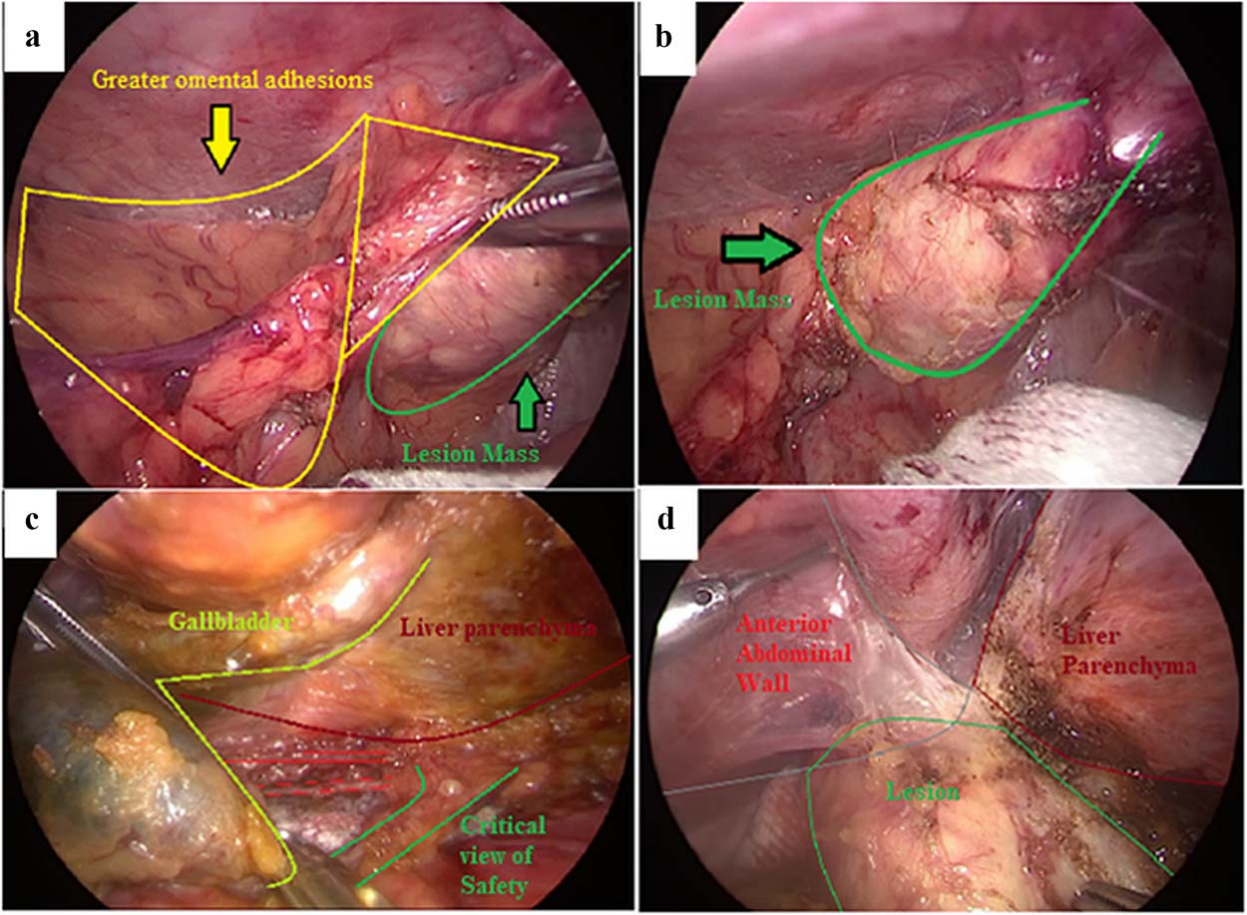
Intraoperative laparoscopic findings during cholecystectomy. (a) Dense inflammatory adhesions involving the greater omentum with an associated lesion mass at the gallbladder fundus. (b) Close-up view of the firm lesion mass at the fundus (outlined). (c) Dissection along the gallbladder–liver interface to obtain the critical view of safety. (d) Calcified lesion adherent to the anterior abdominal wall near the liver margin, excised en bloc with the gallbladder.

Laparoscopic exploration revealed extensive diffuse peritoneal adhesions despite no prior abdominal surgery (Fig. 2a). Dense inflammatory adhesions involved the gallbladder, duodenum, small bowel, and greater omentum, and a firm hard mass was present at the fundus (Fig. 2b). Dissection was performed close to the gallbladder serosa to separate it from adjacent structures. The critical view of safety was achieved (Fig. 2c), and the cystic duct and artery were divided. During gallbladder bed dissection, a calcified mass adherent to the anterior abdominal wall near the liver margin was identified and excised en bloc with the gallbladder (Fig. 2d).

**Figure 3:**
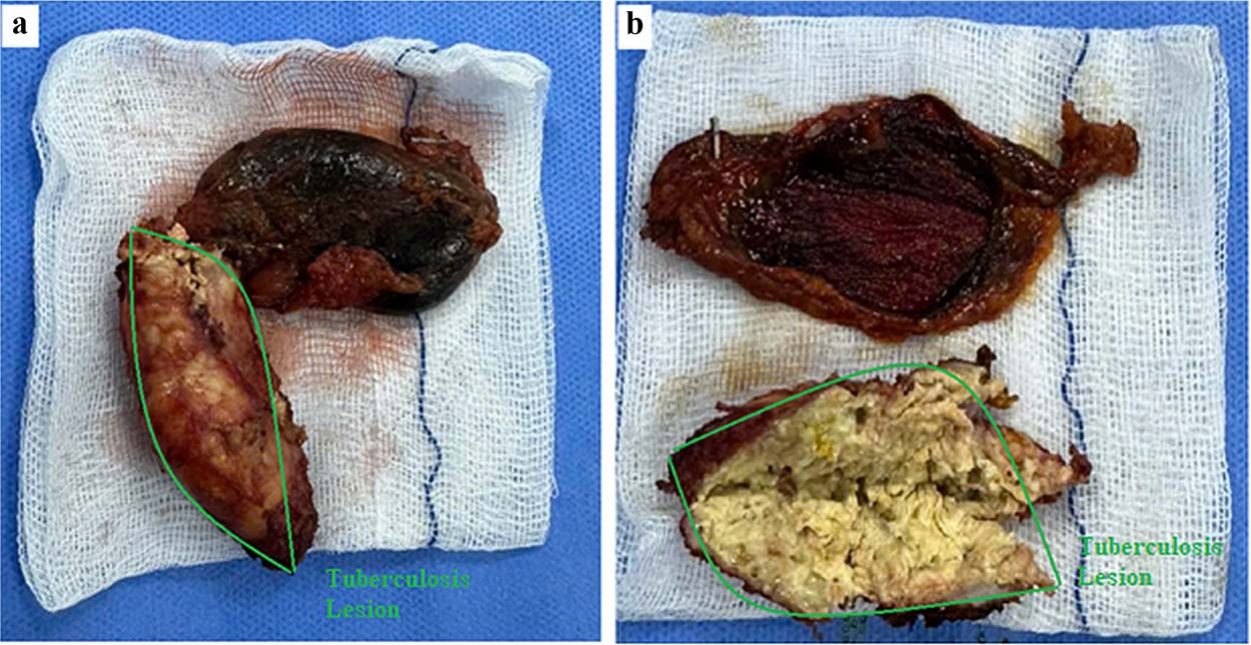
Gross specimen of the gallbladder with tuberculous calcified lesion. (a) Resected gallbladder with a hard calcified structure continuous with the fundus (outlined). (b) Sectioned specimen showing thick whitish caseous-like material within the lesion, consistent with caseating granulomatous inflammation.

**Figure 4:**
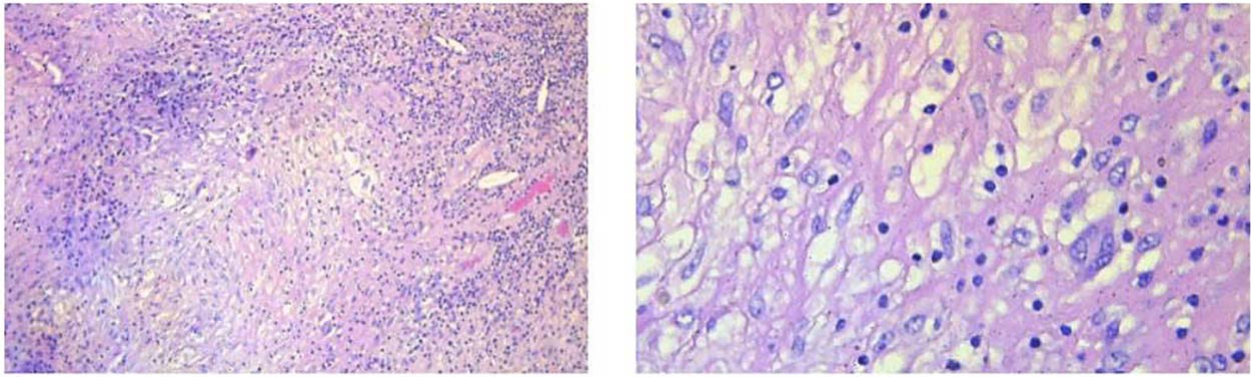
Typical tuberculous granuloma with central caseous necrosis surrounded by epithelioid histiocytes and Langhans-type giant cells.

Gross examination revealed a hard calcified structure continuous with the gallbladder fundus (Fig. 3a). Upon sectioning, the lesion contained thick, whitish material resembling caseous necrosis (Fig. 3b). Intraoperative frozen section demonstrated granulomatous inflammation with caseous necrosis, consistent with GBT. Final histopathology confirmed typical tuberculous granulomas with Langhans giant cells located beneath the epithelium (Fig. 4). Based on these findings, no further surgical intervention was performed. A subhepatic drain was placed prophylactically.

The postoperative course was uneventful. The patient passed flatus on postoperative day 2, experienced minimal pain, and laboratory tests and postoperative ultrasonography were normal. The drain was removed on postoperative day 3, and the patient was discharged on postoperative day 4. At two-week follow-up, no complications were noted. The patient was subsequently referred to a specialized tuberculosis center to initiate antituberculous therapy.

## Discussion

Vietnam remains a high-burden tuberculosis country, with extrapulmonary tuberculosis accounting for approximately 15%–20% of cases. Among these, abdominal tuberculosis represents about 12%, while hepatobiliary involvement constitutes only around 1% [2]. GBT is even rarer and was first described in the literature in 1870. Its rarity is thought to be related to the alkaline nature of bile, which provides a hostile environment for *Mycobacterium tuberculosis*, rendering gallbladder involvement uncommon and often associated with disseminated disease. *Mycobacterium tuberculosis* may reach the hepatobiliary system via hematogenous spread, lymphatic dissemination, or direct extension from adjacent organs [2]. Local factors, such as gallstones or cystic duct obstruction, may compromise gallbladder defenses and facilitate infection [6], which is supported by reports indicating that up to 60% of patients with GBT have concomitant cholelithiasis [2].

Clinically, GBT lacks specific features and commonly presents either as cholecystitis-like inflammation or as a tumor-like mass, the latter posing the greatest diagnostic challenge due to its close resemblance to gallbladder carcinoma [2]. Calcification of the gallbladder, regardless of pattern, is widely regarded as a radiological feature suggestive of malignancy, further complicating preoperative diagnosis [3, 5].

Radiological findings are nonspecific and may include wall thickening, nodules, or mass lesions [4]. Although features, such as central necrotic lymph nodes, may suggest tuberculosis, these findings are often indistinguishable from malignancy [4]. Histopathology remains the gold standard for diagnosis, typically demonstrating caseating granulomas with Langhans giant cells [4, 7, 8].

Intraoperatively, distinguishing GBT from carcinoma is equally challenging, as both may present with thickened, rigid gallbladder walls, dense adhesions, hepatic infiltration, and enlarged hilar lymph nodes [2, 7, 8]. Frozen-section biopsy plays a crucial role in avoiding unnecessary radical surgery. In our case, the patient was asymptomatic with normal tumor markers, yet imaging and operative findings strongly suggested malignancy. The use of intraoperative frozen section was pivotal in preventing overtreatment. The severe adhesions observed may represent subclinical disseminated abdominal tuberculosis.

## Conclusion

Gallbladder tuberculosis should be considered in the differential diagnosis of calcified gallbladder masses, particularly in tuberculosis-endemic settings and in patients with prior tuberculosis. Awareness of this mimic and judicious intraoperative histopathological assessment can prevent overtreatment and optimize patient management.

## Data Availability

All data sets collected during this study are available upon reasonable request from the corresponding author.
